# CO_2_ Laser-Based Rapid Prototyping of Micropumps

**DOI:** 10.3390/mi9050215

**Published:** 2018-05-03

**Authors:** Zachary Strike, Kamyar Ghofrani, Chris Backhouse

**Affiliations:** 1 Electrical and Computer Engineering, and Waterloo Institute of Nanotechnology, University of Waterloo, Waterloo, ON N2L 3G1, Canada; zack.strike@edu.uwaterloo.ca; 2 DropLab Inc., 151 Charles Steet West, Kitchener, ON N2G 1H6, Canada; kghofrani@edu.uwaterloo.ca

**Keywords:** micropumps, microvalves, rapid prototyping, CO2 laser ablation, microdroplets

## Abstract

The fabrication of microdevices for fluidic control often requires the use of flexible diaphragms in a way that requires cleanroom equipment and compromises performance. We use a CO${}_{2}$ laser to perform the standard ablative techniques of cutting and engraving materials, but we also apply a method that we call laser placement. This allows us to fabricate precisely-positioned and precisely-sized, isolated diaphragms. This in turn enables the rapid prototyping of integrated multilayer microfluidic devices to form complex structures without the need for manual positioning or cleanroom equipment. The fabrication process is also remarkably rapid and capable of being scaled to manufacturing levels of production. We explore the use of these devices to construct a compact system of peristaltic pumps that can form water in oil droplets without the use of the non-pulsatile pumping systems typically required. Many devices can be fabricated at a time on a sheet by sheet basis with a fabrication process that, to our knowledge, is the fastest reported to date for devices of this type (requiring only 3 h). Moreover, this system is unusually compact and self-contained.

## 1. Introduction

Micropumps and microvalves form the basis of many microfluidic applications and are the subject of a number of reviews [1,2,3,4]. Mechanical displacement micropumps (MDMs) are particularly versatile, and peristaltic pumps made from three linked microvalves [1] are readily integrated into microfluidic technologies. From the early microvalve designs (e.g., [5,6]), such valves and pumps have often been based on the use of a single uniform sheet of the diaphragm material, often of polydimethylsiloxane (PDMS), selectively bonded to a more rigid substrate such as glass or silicon. However, materials such as glass and silicon are expensive as compared to polymers, and their fabrication methods tend to rely on cleanroom processes.

Polymeric materials have become increasingly important in microfluidics (e.g., [7,8]), and the role of thermoplastics has the potential to greatly facilitate both research and manufacturing. A number of reviews has described thermoplastic microfabrication methods [7,8,9,10,11,12,13], and recent work [14] discussed the transition from glass and silicon to thermoplastics, while discussing the available fabrication methods for these materials, methods that are often far faster and less expensive. As recently surveyed [15,16], a wide range of demonstrations has developed the use of thermoplastic substrates especially (e.g., [17] and the references within) in integrations of PDMS with thermoplastics such as poly(methyl methacrylate) (PMMA) and using methods such as plasma activated bonding and hot embossing. Very low cost fabrication methods have been emphasised for such membrane-based chips, for example the recent work [18] that thermally bonded layers of a variety of polymers to form microvalves. Laser-based fabrication has become a powerful technique for the production of microfluidic devices [7,8,19], and the 10.6 μm emission of CO${}_{2}$ lasers provides an effective way of patterning materials, particularly polymers and oxides. From its initial demonstration [20] in application to PMMA, CO${}_{2}$ laser patterning has been applied to a very wide range of microfluidic materials such as glass [21], quartz [22], PDMS [23], polytetrafluoroethylene (PTFE [24]), polystyrene (PS [25]), polycarbonate (PC [26]), polyester/polyethylene terephthalate (PET [27]), cyclic olefin copolymer (COC [28]), paper [29] and laminates, both polymeric [30] and co-fired green ceramics [31].

It has been noted that unlike many thermoplastics, PMMA is ideal for use with CO${}_{2}$ lasers as it vapourises effectively [32] and forms a clean cut. The use of CO${}_{2}$ lasers was presented as an ideal way of enabling rapid prototyping of PMMA [33]. CO${}_{2}$ laser-based microfabrication with PMMA has been used in a wide range of applications, from droplet formation [34], to cell culture [15] and electrophoretic devices [35]. Rapid prototyping methods for microfluidics have been compared [36], but laser ablation appears to be a preferred method, with the CO${}_{2}$ laser-based processing of PMMA being perhaps the most commonly-used approach. The combination of laser ablation followed by thermal bonding can be remarkably versatile and effective, as demonstrated recently [26] in applications to PMMA, PET, PC and PS.

The relationship between the laser ablation conditions and the ablated parameters of the resulting PMMA features (e.g., depth and width) has been well studied (e.g., [20,32,37,38,39]). The smoothness of the channels has been studied (e.g., [37,39]) and found to be improved by the use of a relatively unfocused beam [33], by heating during laser processing [40], by multiple passes of the beam [41,42] or by solvent exposure [41,43].

The type of fabrication technology constrains the ability to place isolated diaphragms. As examples, the lamination of CO${}_{2}$ laser-cut layers was shown to be a very effective means of rapid prototyping [44] (taking on the order of 24 h), and the alignment of layers was accomplished by means of a registration frame. In a similar fashion, xurography has been used to mechanically cut out layers for subsequent lamination and use as PDMS moulds [45]. Neither approach could be used to position an array of small (isolated) circular membranes for use in the microvalves. To our knowledge, there have been no reports of a way of positioning an isolated region of releasable diaphragm in this way.

We note that although laser welding has been applied to polymeric substrates, this is often done in a transmission mode, i.e., with a visible wavelength and an absorbing ‘nanolayer’ (e.g., [46]). Laser edge welding has been demonstrated with a CO${}_{2}$ laser (without an absorbing ‘nanolayer’) to weld the edges of a chip together [47]. Past experimental and simulation work (e.g., [48,49]) indicate that mm-scale (in depth and width) welds should be feasible for adequately thick (typically > 30 μm) polymer layers. This suggests that the selective placement of membranes by welding a thin (e.g., 10 μm-thick polymer layer within a small (mm-scale) feature may be be problematic.

Recent reports [15,16] demonstrated a very effective way of rapidly fabricating diaphragm-based PMMA and thermoplastic polyurethane (TPU) microfluidics. Rather than use cleanroom-based photolithography in a complex process (typically several days or more), these recent reports used a CO${}_{2}$ laser to pattern thermoplastics, forming arbitrarily complex microfluidics within about a day and without cleanroom equipment. However, these methods still used an approach that is intrinsically highly heterogeneous and requires significant manual intervention during fabrication, thereby limiting the scalability of the fabrication process.

We have explored the use of an automated laser-based method of placing portions of a film in a layer by layer assembly process that involves fusion bonding and adhesive tapes. Recent reviews of laser-based additive methods for MEMS have tended to emphasise methods such as the laser sintering of powders or laser-assisted forward transfer of portions of films (e.g., [50]) without any reports of methods similar to what we have explored. The closest we have encountered are the computer-aided manufacturing of laminated engineering materials (CAM-LEM) method [51] or a method used in composite manufacturing (e.g., the automotive industry) of the so-called laser-assisted tape placement (LATP) method [52,53]. The CAM-LEM method is the one used to construct layered structures of laser-patterned ceramics and metal, while the LATP method [53] uses a laser to pattern a tape and also to assist in fixing it in place. We are not aware of any laser-based method for the precise positioning of a release layer in microfluidic applications.

Of particular importance to pumping and valving applications is the use of elastomeric materials. However, in microvalve and micropump designs, this often leads to the elastomer forming a large proportion of the active surface of the device (e.g., forming the top layer of a microchannel), and this leads to significant heterogeneity in materials’ properties. Although this has been used to advantage by, for instance, allowing oxygen exchange through a relatively permeable TPU membrane [15], this heterogeneity can lead to undesired variations in biological and chemical compatibility, zeta potential, contamination and reagent loss due to permeability. In addition, the placement of the diaphragm material may also require manual intervention, whether to precisely position the diaphragm itself, or to do so for a localised layer of another material in order to ensure that the diaphragm is able to release from the surface (e.g., the manually-placed spot of a marker pen at each valve [16,54,55]). Ideally, one would have the ability to minimise the amount of heterogeneity and maximise the choice of potential materials while not requiring any device-level manual intervention for the placement of the release layer.

**Figure 1:**
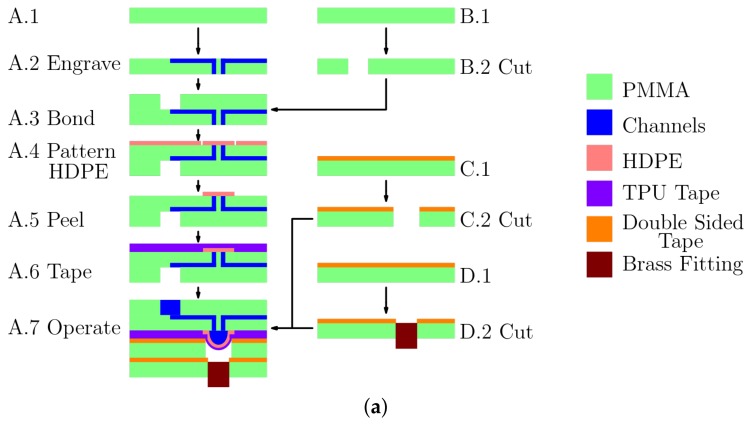

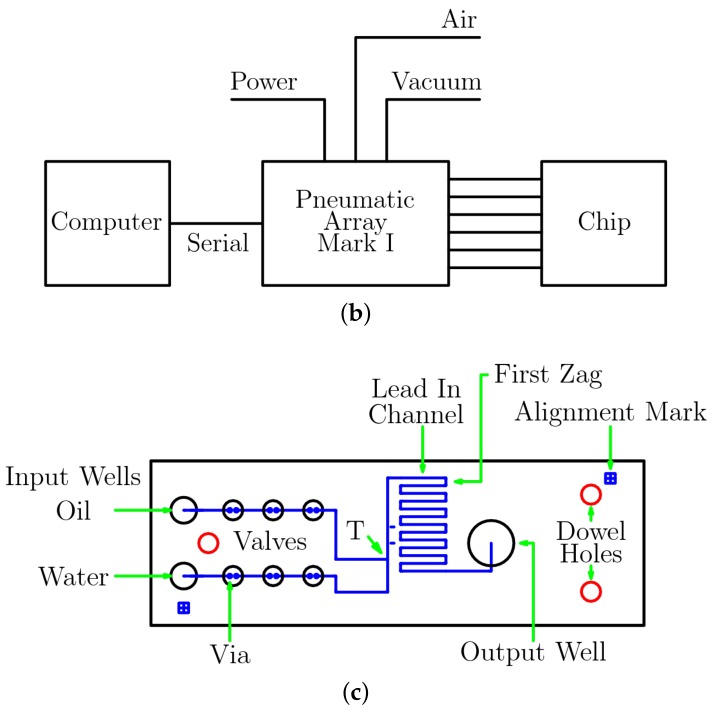
(**a**) The fabrication process cuts and pattern pieces of PMMA: one with channels and vias (A.1), and another with cut-outs alone (B.1). The pieces are engraved, cut and thermally bonded (Steps 2, 3), and then, a layer of HDPE is applied and laser patterned (A.4, A.5). TPU tape is then applied (A.6), and two capping layers are then added using double-sided tape. The outermost capping layer contains a brass fitting that is directly connected for pneumatic actuation. (**b**) As depicted, the overall system consists of a commercially-manufactured pneumatics control system (Pneumatic Manifold Mark I, a shoe-box-sized instrument from Droplabs) that connects directly to the chip itself and to a laptop computer via a serial cable. (**c**) A top-view of the chip design (76.2 mm × 25.4 mm) containing microchannels (blue lines), three wells (larger black circles), 12 vias (blue dots) within six microvalves (smallest black circles) that form the two peristaltic pumps. The microchannels link the reagent reservoirs (input wells, one of oil, one of coloured water) to the pumps and a T-intersection followed by a zig-zag structure and a waste well (output well). The design also includes dowel holes (red circles), which are used to align the layers of the chip during fabrication.

Although a very wide range of PDMS-based methods have been reported, the use of Viton as a more robust elastomer was introduced [55] for microfluidics based on hot embossing, plasma activation and chemically-assisted fusion bonding. Similar work explored the use of styrenic thermoplastic elastomers [56]. The use of polyurethane as a diaphragm was found [57] to be an effective substitute for PDMS, one that is more manufacturable and more robust. The recent demonstrations of fusion-bonded PMMA and TPU membranes [15,16] have shown a remarkably effective rapid prototyping method. We have extended that work by (1) using vias to minimise the amount of heterogeneity and (2) using a method of laser placement so that membranes are automatically placed where needed. Figure 1a shows the cross-section of the technology (not to scale, described in more detail in the Materials and Methods Section). Although we might have used the recently-reported approach [15] of fusion-bonding a layer of TPU to the PMMA, we found that the use of an adhesive TPU tape allowed for an effective (and fast) means of fabrication, taking less than 3 h of elapsed time (1 h of labour) for a fabrication cycle.

Our method is a surprisingly rapid one for fabricating diaphragm-based microfluidics. We used this new fabrication method to explore a fundamental drawback of MDMs: that they move fluid in discrete steps, i.e., the flow is intrinsically pulsatile, and this can limit their use. Since droplet generation (e.g., water droplets in an oil carrier) is easily disrupted by pulsatility (i.e., variations in the pressure), MDMs are not commonly used for microfluidic droplet generation. In fact, demonstrations using MDMs to date have generally used non-pulsatile pumps (typically off-chip syringe pumps) to drive one or both of the phases [58,59,60], and this is a great disadvantage. In the present work, by integrating two peristaltic pneumatically-actuated MDMs, we successfully demonstrated water-in-oil droplet methods in a fully-integrated chip, without the requirement of any additional pumps.

## 2. Materials and Methods

Extruded PMMA, nominally 0.06 inches thick (actual thicknesses 1.53 to 1.56 mm), Optix brand, was purchased from Plaskolite. Two types of tape were used, polyurethane tape (TPU tape: 3M 8672, McMaster Carr S-16236, adhesive on one side and 200 μm thick) and double-sided tape (3M 442 KW, 100 μm thick). Release layers were formed from sheets of ‘Compliments Plastic Wrap’-food wrap with low permeability ≈10 μm thick and believed to be high density polyethylene (HDPE) or similar. Deionized water and mineral oil (M5904, Sigma-Aldrich, Oakville, ON, Canada) were used while testing flow rates and for droplet formation. Then, 2 wt % SPAN 80 (S6760, Sigma-Aldrich, Oakville, ON, Canada) was added to the mineral oil to stabilize the water-oil interface. Consumer-grade blue food colouring (Clubhouse Neon, McCormick, London, ON, Canada) was added to the water phase to increase the visibility of water in the channels and to provide additional contrast between water and oil. Photographs were taken with a stereo microscope using a 9-megapixel camera (Model MU900, AmScope, Chino, CA, USA) using Toupview software. The video (at 13.7 frames per second) of the resulting droplets was inspected frame by frame, and the position of fluid fronts measured. Full chip images were taken with a Nikon D3200 with an AF-S NIKKOR 18–55-mm lens (Tokyo, Japan). A Universal CO${}_{2}$ laser system (Model# VLS 2.3 with a 30 W laser tube and 2-inch HPDFO focusing optics) was used to pattern the microfluidic chips using laser ablation with an engraving speed of ≈ 0.07 m/s. A forced air convection oven (Fisher Sci. Model #6916) was used for thermal bonding. Bulldog clips (Staples, Part #671985) and glass microscope slides were used to clamp the PMMA layers together during the bonding. Tygon tubing was used to connect to the chip with 41-cm lengths, each with an inner diameter of 1.59 mm. Positive pressures were provided by an SMC AR20-N02BG-Z-A regulator (0.05 to 0.67 MPa with indicator markings at 20-kPa intervals) connected to the building supply of clean-dry air, which was used to supply 25 kPa to 100 kPa with an estimated accuracy of ± 10 kPa. Negative pressures were provided by an SMC IRV10-N07BG regulator (−1.3 to −100 kPa with indicator markings at 10-kPa intervals) connected to the building vacuum line, which was used to supply −15 to −60 kPa with an estimated accuracy of ± 2 kPa.

### 2.1. Fabrication

Microfluidic chips were designed with Asymptote (a descriptive vector graphics language [61]) to generate a postscript file that was input into CorelDraw X5 (Corel, Ottawa, ON, Canada) and printed to the laser using a proprietary driver. The chips were fabricated by patterning the features into the PMMA (e.g., Steps A.2 and B.2 shown in Figure 1a using laser ablation with the parameters given in Table 1 and with the long side of the chip in the left-right (*x*-axis) direction. Narrow holes (vias, about 160 μm in diameter) were laser-drilled through in Step A.2 using ten passes of the laser. The wells and dowel holes were cut out in the same step.

After patterning, PMMA layers were then washed in water with detergent (Palmolive Original), well rinsed with de-ionised water and blown dry with an air gun.

The pieces cut in Steps A.2 and B.2 were aligned by edge registration and eye using the alignment marks (Figure 1c) on each piece (The alignment marks consist of a square (1.5 mm × 1.5 mm on the upper pattern, 3 mm × 3 mm on the base pattern), with a cross going through the centre of the square. The alignment marks were designed to overlap when properly aligned.). The pieces were then clamped together between glass microscope slides and thermally bonded (Step A.3). The clamping action was provided by bulldog clips, and the assembly was placed in a preheated 115 ${}^{\circ }$C oven for 30 min whereupon the oven was set to 80 ${}^{\circ }$C for 1 h, after which the oven was turned off and left to cool down to 60 ${}^{\circ }$C or less (≈30 min). A layer of HDPE was placed atop the fusion-bonded layers, and a segment large enough to cover all the valves was cut out, leaving a single sheet of HDPE atop the bonded layers. Then, using the dowel holes, these were then placed in an alignment jig in the laser system such that the laser could be used to automatically cut out small circles of HDPE around all the vias (Step A.4). A single peeling operation of the HDPE sheet leaves behind a large number of accurately-positioned, isolated structures (membranes). Each of these circular membranes (6 for each chip) is surrounded by a moat, a trench from the cutting operation. This moat is not connected to any other fluidics. A TPU tape was then applied (Step A.6) over the HDPE circles, sealing them in place, but allowing deflection over the vias, thereby forming circular diaphragms above each pair of vias. A manifold with circular holes was then cut out from a sheet of PMMA to which one side of a double-sided tape has been affixed. This was then attached directly to the TPU tape covering the vias, forming circular diaphragms above each pair of vias. Brass fittings (Clippard Instrument Laboratory Inc., Cincinnati, OH, USA) 11750-2 3-56 to 1.59-mm ID Hose Fitting) can be screwed directly into the manifold using Viton 1 mm × 1.5 mm O-rings to provide a good seal. Although these two capping layers (C and D) could be combined, by using two layers, we can readily change the valve diameter while keeping the brass fitting unchanged. Following bonding and assembly, the alignment was verified by photographing the alignment patterns of each chip, allowing the alignment to be determined with a measurement accuracy estimated at ±30 μm.

### 2.2. Operation

The computer-controlled pneumatic system was similar to that described in our past work [54], but can be controlled with more precise timing given the integrated microcontroller that takes commands from a computer connected via a universal serial bus (USB) link. The pumps can be operated independently and are configured so that only one is operating at a time. A pause time after each step can be specified, and values of 0.06, 0.15 or 0.3 seconds were used, corresponding to pump frequencies of 2.5, 1.0 and 0.5 Hz, respectively. The frequency is the inverse of the time taken to run through the six steps required to complete a pumping sequence for each of the two pumps (Table 2).

Before forming droplets with a chip, the aqueous pump was cycled (with the third valve of the oil pump closed) until the water reached 2.5 mm past the T-Junction. The oil pump was then cycled, with the third valve of the aqueous pump closed, until the entire channel past the T-junction was filled with oil. After those steps were performed, the chip was operated with the pumping sequence in Table 2 for three min, such that droplets could be formed and fill the chip. During this time, the pressure settings on the regulators were adjusted to achieve the optimal pressure settings. Then, the chip would be tested with the pumping sequence in Table 2 and the pressure settings adjusted for optimal droplet formation. The chip was then operated for one minute before any videos or pictures were taken of it. On occasion, some valves were not immediately usable. In this case, a pressure of −20 kPa was applied for as much as 5 min to release the membranes, after which the valves operated normally.

### 2.3. Measurement

The laser cut substrates can readily be cleaved along the line of the channel and imaged under an optical microscope, allowing the simultaneous measurement of the depth and width of the channels, using the 1.5-mm substrate thickness as a reference width. The droplet sizes were measured in two different ways, from video frames in a fixed location, but varying time, and by measurement from a still high-resolution image (spatial variation, but fixed time). In order to cover large areas (larger than the microscope field of view) with several still images, we used ImageJ [62] with software to stitch images [63] as combined in the Fiji package [64]. Gimp (under Ubuntu 16.04) was used to adjust the contrast and brightness of images for better readability.

## 3. Results

**Figure 2:**
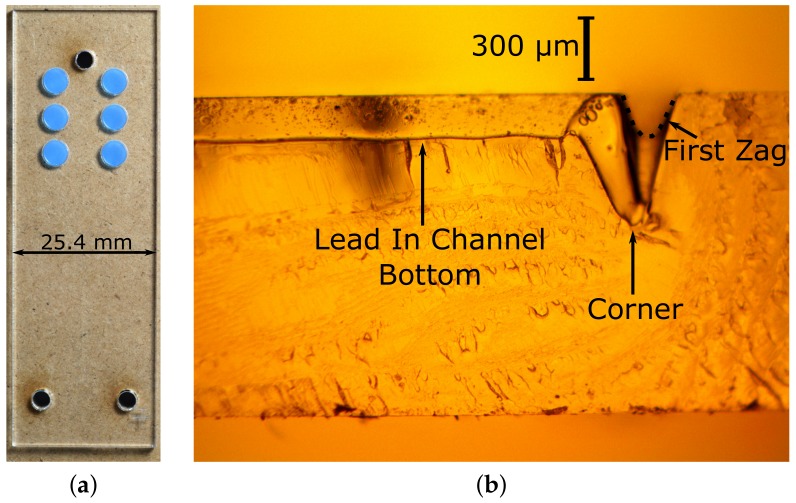
(**a**) As a demonstration of the technique of laser-placement, small circles were patterned by laser in a sample of as-received PMMA that is coated in a layer of blue protective plastic. The blue plastic layer was then peeled off leaving the isolated blue circular membranes in position. A single peeling can prepare a large sheet of an arbitrarily many circles (the release layers actually used were transparent HDPE held in place by van der Waals forces). (**b**) A cross-section of the microchannel leading up to the zig-zag section (the horizontal section is the lead in; the start of the zig-zag goes into the page on the left, outlined by a dashed black line). Additional laser processing of the corner itself has deepened it substantially; the depth is about 200 μm in the lead-in channel, increasing to (very briefly) 640 μm, and (going into the page) to 550 μm before decreasing to 200 μm in the ‘zag’ channel (without this additional laser processing, the channel corners tended to contain narrowed sections).

As shown in Figure 2a, the technique of laser placement was found to be effective, whether with the demonstration with the blue protective tape (shown in Figure 2a) or when used with the thin film of clear HDPE. As shown in Figure 2b, the laser processing leads to channels that were Gaussian in profile and roughly semicircular, about 200 μm deep and 210 μm wide, although the corners suffer a very significant variation in cross-section (several times deeper). The channel dimensions vary slightly (and smoothly) by about 10% along the length of the cuts due to some combination of the laser driver software, laser parameters and firmware and the chip design. These variations do not appear to affect operation, and all channel dimensions should be considered to be accurate to within 10%. Due to a slight astigmatism in the laser profile, top-down microscope images show a slight difference between the apparent width of channels cut along the *x*-axis (left-right in the laser, corresponding to the long axis of the chip) and those of the *y*-axis (e.g., Figure S1). However, the astigmatism seems to be in the edges of the beam, hence affecting the shallow edges of the channel. Channel cross-sections in the two directions with similar depths (e.g., the depths of the lead in channel and the orthogonal zag channel of Figure 2b) have the same depths to within our uncertainties).

Mineral oil (clear, as the continuous phase) and water (blue, as the dispersed phase) were pumped using the two on-chip pumps. We found the effective ranges of negative pressure to be between −2 and −60 kPa, while the system seems relatively insensitive to the range of positive pressure. The range of actuation frequency could be arbitrarily slow and as fast as about 2.5 Hz. The overall flow velocity could be as high as about 2 mm/s with volumetric flow rates between 25 and 125 nL/s. The droplets could be arbitrarily large and as small as about 0.4 mm (≈ 30 nL). The optimal conditions were found to be with 1 Hz of operation with positive and negative pressures of 40 and −22 kPa, respectively, where successive droplets varied in size by ≈ 5%. There were significant day to day variations in droplet size (about 25%), apparently due to differences in the pressure setting (due to readout uncertainty). There were also significant variations of droplet size with position, apparently due to variations in the channel cross-section (notably at the corners).

**Figure 3:**
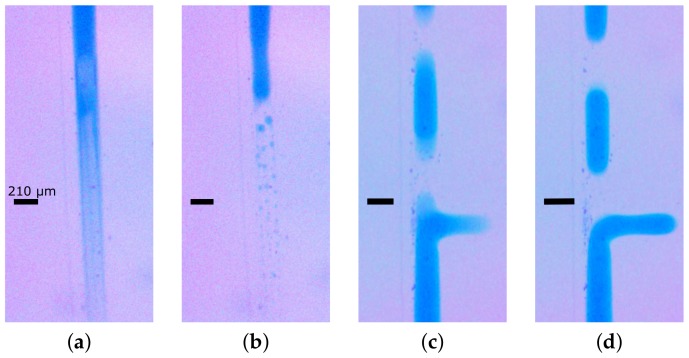
The transient effects of the pumping cycle can be seen in successive video frames to have a large effect on the stability of the droplets that can be formed with oil/water flows. In all images, the width of the channels is the same, 210 μm, and a scale bar of this size is shown in each image. (**a**) Shows a blue water droplet just after being moved in a pump cycle where the light blue smear below the droplet is thought to represent the long sheath of coloured water left behind in a step of pressure-driven flow under conditions of high positive pressures (80 kPa). (**b**) One frame later, this large and substantial sheath is seen to have coalesced into discrete satellite droplets that trail the primary droplet. (**c**) Shows a blue water droplet just after being moved under optimal conditions. The gentler pump cycle formed a shorter and less extensive sheath of trailing coloured water. (**d**) One frame later, this smaller and less substantial sheath is seen to have retracted into the primary droplet.

The pneumatic system was used to explore the magnitude of the pulsatility under various conditions. Since we could not directly measure the pressure within the chip, we measured how much the droplets moved backwards during the pump cycle, although the droplets would on average move in one direction, they would, at one step of the pumping cycle, move backwards. We refer to this as the backstep length. The backstep was found to be highest (about 60% of the droplet length) when using large magnitudes of positive and negative pressures (e.g., −60, 80 kPa) and slow stepping (e.g., 0.5 Hz), but under the gentler optimal conditions (with at least 1 Hz), the backstep was reduced to about 20% of the droplet length. It was also found that this directly affected the stability of droplets being moved on chip. Figure 3a shows that for pulsatile flow (i.e., large backsteps), the droplets suffer an extended smearing after each step. Although much of this smeared material was resorbed by the droplet, much of it coalesced into a train of much smaller and polydisperse droplets, as shown in Figure 3b. We refer to this phenomenon as a comet tail. By contrast, under optimal conditions the amount of smearing was far less (Figure 3c), and this appears to be fully resorbed without forming any satellite droplets, i.e., the droplets remain intact.

**Figure 4:**
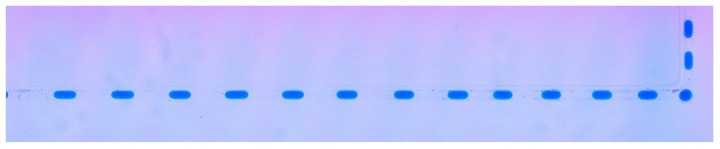
Under optimal operating conditions (P${}_{+ve}$ = 40 kPa, P${}_{-ve}$ = −22 kPa, 1 Hz), droplets can be formed with a length of 0.40 mm ± 0.02 mm (measured from video footage as each droplet leaves the intersection area). A train of droplets is generated at the T intersection at the far left and moves into the zig-zag section as it leaves the figure (the droplets had been generated for 1 min prior to taking these images). The length from the intersection to the first corner (i.e., from left to right) is 12.5 mm, and due to a limited field of view, this image was formed from several individual pictures. There is a slightly smaller appearance of the droplets on the right, and this appears to be due to variations in the channel size.

Figure 4 shows a train of droplets generated by the chip of Figure 1c. The droplets appear to be slightly smaller on the right of the image, and this is a consistent feature. A series of linear fits of droplet size vs. distance showed a shortening of the droplets as they move to the right in this channel, typically by about 20% over the length of the channel (data not shown). We hypothesise that this is due to a slight deepening of the channel from the laser spot decelerating as it nears the corner (hence, a higher energy density and more ablation). In addition, the corners are deeper due to the intersection of vertical and horizontal channels, and hence, the droplets tend to be appear very significantly smaller as they pass through the corners (we also expect that the droplet length will vary slightly depending on the number and size of droplets downstream in the channel since more droplets will lead to lower oil content, lower flow resistance and, hence, to slightly longer droplet lengths). These topics are under continued investigation.

In this type of application, leakage is not a primary concern given that the maximum pressure differences driving the fluids through the pumps (e.g., when sealed) is about 10 Pa, corresponding to perhaps 1 mm of height difference. However, tests of liquid leakage were made by tilting the chip to create a 10-mm difference in height between the oil and water wells, and the rightmost valves were sealed. After 30 min, the fluids (mainly the oil) had moved by approximately a droplet length, corresponding to about 15 fL/s. This phenomenon may be controllable by increasing the positive pressure. However, these leakage rates would not affect normal operation in droplet formation. This is a topic of ongoing investigation. No leakage of air through the diaphragm was observed even under prolonged pneumatic actuation with positive pressure (i.e., no bubbles were seen).

**Figure 5:**
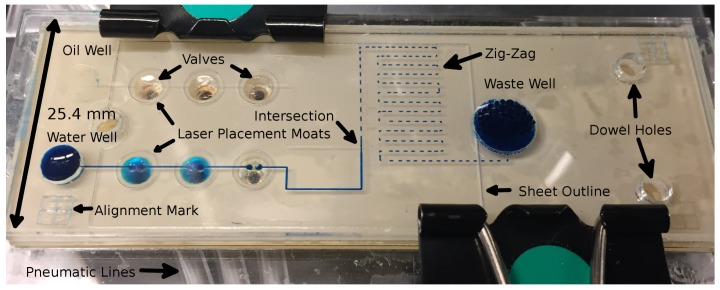
Under the conditions of minimal backstep, the chip (with features as defined in Figure 1c) generates a uniform train of droplets of water (blue) in oil (clear) that are drawn from the oil and water reservoirs on the left and marched through a complex structure to a waste well on the right. The chip is 76.2 mm × 25.4 mm, and underneath are the six brass fittings screwed into the base of the chip, each connecting to an output from the pneumatic controller, which is in turn controlled via a laptop computer. The laser placement moats and sheet outline are from the laser placement of the HDPE membranes and are not connected fluidically. The pneumatic tubing can be seen underneath the water valves. The yellow appearance of some of the valves is due to reflected light from the brass pneumatic fittings.

Figure 5 shows a train of droplets present throughout the fabricated chip. Under these optimal conditions, the droplets remain much the same size, although we see some shifts in droplet spacing as the droplets go around corners where the deeper portions of the channel allow redistribution of the oil between the droplets. Nevertheless, the droplets are essentially uniform until the first corner.

## 4. Discussion

We have used the method of laser placement to build an integrated pair of micropumps and used this to explore the use of gentle (non-pulsatile) pumping for droplet formation. We have demonstrated the formation and manipulation of a uniform stream of microdroplets and have done so in an unusually compact system. The system does not require the syringe pumps normally used in conjunction with this application and has integrated all the MDMs within a single microscope slide-sized device that is operated by a single low-cost pneumatics manifold and a laptop computer.

The fabrication procedure has misalignments of less than 30 μm (i.e., small enough not to jeopardise functionality). As shown in the Supplementary Information (e.g., Figure S2), earlier work had larger alignment variations, and initial experimentation used relatively large pressure differences (e.g., negative pressures to −60 kPa). Negative pressures of this magnitude seem to have led to the deformation of the HDPE and the formation of wrinkles in the film (also apparent in Figure S2a) that do not obviously affect the operation of the chip. Some combination of alignment error and excessive pressures may have led to a leakage of blue fluid into the moat surrounding the release layer (see the bottom right valve of Figure S2a). With better alignment and when only ever used at the optimal conditions, wrinkling is not readily apparent, and such leakage into the moat did not occur (Figure S2c). Future work might explore the use of thinner HDPE (for less wrinkling) or to increase the diameter of the release layer (to avoid further leakage). It is not clear that this will be needed.

The dry as-fabricated channels (i.e., prior to contacting water) are shown in Figure S1a, showing no major particles of laser debris. The main issue affecting reliability appears to be the characteristics of the channel where the laser cut starts, stops or joins another channel, i.e., the channel depth varies significantly at these three points, as shown in Figure 2b. The control of this variation is a topic of ongoing development. Following contact with water droplets that have been stationary, it would appear that water is trapped in pockets in the walls (as apparent in Figure S1b). However, preliminary measurements suggest that the stationary droplets are about 10% wider than the moving droplets. We attribute this to a sheath of oil around droplets moving in these chips. We are exploring the possibility that as long as the droplets are kept moving, they will not contact the walls.

As discussed in the Supplementary Data (Figure S1) of a recent publication [15], the change of focus height can be used to change the channel profile substantially over a range of several mm. The present work uses a relatively small offset that does not clearly change the beam profile, but might produce slightly sharper vias.

At the fabrication level, it is useful to compare and contrast the present work with very recent reports by others [15,16]. That remarkable work used a rapid prototyping approach that had great similarities to the present work, as well as major differences. In the present work, the use of extruded PMMA in laser processing led to smoother channels that did not require the use of the chloroform smoothing used in those reports (where cast PMMA was used). This also allowed us to avoid the subsequent baking step to remove residual solvents, greatly streamlining the process. We did test the effects of sonication, and although the channels appeared slightly clearer (data not shown), their performance was not noticeably improved, so sonication was not made part of the fabrication process of the present work. In addition, our fusion bonding does not involve the use of solvents, also producing a simpler bonding process. The bond strength was sufficient that the layers bonded in Step A.3 never delaminated unless intentionally torn apart. When forcibly delaminated, the interface was clearly weaker than the remainder of the chip, but was easily strong enough for normal handling and processing, e.g., screwing in brass fittings.

The present work requires even less fabrication equipment than a recent (and extremely rapid) report [15] (a CO${}_{2}$ laser and a convection oven) and uses a greatly simplified workflow (several hours vs. more than a day). Although their use of TPU-PMMA fusion-bonding is very appealing, their approach leads to systems with far more surface heterogeneity and less control of the material properties. For instance, heterogeneity in parameters such as the zeta potential and vapour permeability is likely to be disastrous in applications such as integrations of genetic amplification and analysis [54] (in the present work, we have minimised heterogeneity by the use of vias and by using small isolated diaphragms). This is not to denigrate that recent work [15]; in fact, their approach was ideal for their applications (such as cell culture) and has a number of appealing characteristics that we seek to adapt.

In summary, by integrating two on-chip MDMs with a versatile pneumatics controller, we are able to achieve good levels of droplet handling performance without the need for syringe pumps or other off-chip pumps. This integration was based on a greatly simplified rapid fabrication technology for MDMs, one that is significantly faster (and simpler) than previously reported.

## Figures and Tables

**Table 1:** Laser settings for various types of patterning.

Feature	Power (%)	Speed (%)	DPI	Passes	Focus Depth (mm)
Vias	18	25	1000	10	0.7
Channels	18	25	1000	2	0.7
Wells/Through-cuts	20	3	1000	1	1.5

**Table 2:** The two pumps (A and B) each follow the same sequence of steps, but are configured so that each operates its set of three valves only when the other set is not changing (where O represents the valve open and passing fluid, and X represents the valve being closed and sealed). The cycles are completely independent here, but could operate with any degree of overlap or phase shift.

Valve	A1	A2	A3	B1	B2	B3
Step 1	X	O	O	O	O	X
Step 2	O	X	O	O	O	X
Step 3	O	O	X	O	O	X
Step 4	O	O	X	X	O	O
Step 5	O	O	X	O	X	O
Step 6	O	O	X	O	O	X

## Supplementary Materials

The following are available online at http://www.mdpi.com/2072-666X/9/5/215/s1, Figure S1: Droplets in a zig-zag structure, Figure S2: Dry channels and droplets moving within an oil-sheath.

## Abbreviations

The following abbreviations are used in this manuscript:

CAM-LEM	computer-aided manufacturing of laminated engineering materials
COC	cyclic olefin copolymer
HDPE	high density polyethylene
LOM	laminated object manufacturing
LATP	laser-assisted tape placement
MDM	mechanical displacement micropumps
PC	polycarbonate
PDMS	polydimethylsiloxane
PET	polyethylene terephthalate
PMMA	poly(methyl methacrylate)
PS	polystyrene
PTFE	polytetrafluoroethylene
TPU	thermoplastic polyurethane
USB	universal serial bus
