# Supplementary material for: CO2 Laser-Based Rapid Prototyping of Micropumps
Source: Micromachines (Basel). 2018 May 3;9(5):215. doi: 10.3390/mi9050215 (PMC6187535; doi:10.3390/mi9050215)
Supplement: Supplementary file 1 [file micromachines-09-00215-s001.zip › micromachines-284508-SI/logo-mdpi-eps-converted-to.pdf]

MDPI
