# Supplementary material for: CO2 Laser-Based Rapid Prototyping of Micropumps
Source: Micromachines (Basel). 2018 May 3;9(5):215. doi: 10.3390/mi9050215 (PMC6187535; doi:10.3390/mi9050215)
Supplement: Supplementary file 1 [file micromachines-09-00215-s001.zip › micromachines-284508-SI/SupplementaryInformationZSR1.pdf]

Article

CO_2 Laser-based Rapid Prototyping of Micropumps

Zachary Strike [1,†], Kamyar Ghofrani [2,†] and Chris Backhouse [1,*]

1 Electrical and Computer Engineering, and Waterloo Institute of Nanotechnology, University of Waterloo, Waterloo, Canada N2L 3G1
2 DropLab Inc., 151 Charles Steet West, Kitchener, Ontario, Canada N2G 1H6
* Correspondence: Chrisb@uwaterloo.ca; Tel.: +1-519-888-4567 extension 31467
† These authors contributed equally to this work.

Version May 3, 2018 submitted to Micromachines

---

1. Supplementary Data

1.1. *Figure S1: Dry Channels and Droplets Moving Within an Oil-Sheath*

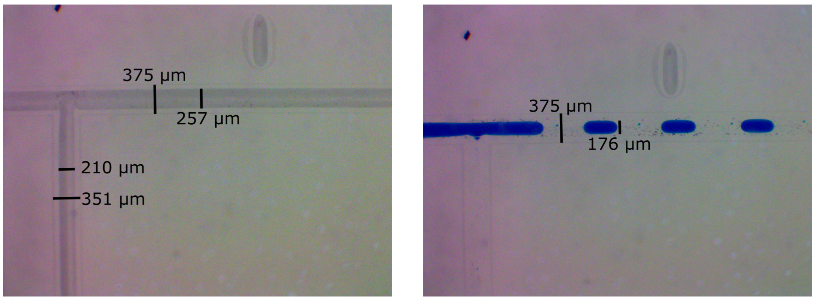

(a) (b)

Figure 1. **(a)** An image of the fabricated chip near the T-intersection prior to any contact with liquid. The slight astigmatism of the laser beam is seen here, producing slightly different channel profiles in the horizontal and vertical directions. In either case two widths are shown, the larger due to the heat-affected zone and the smaller due to the channel itself. (Please note that these images are rotated by 90 degrees from the image of Fig. S1a.) **(b)** A photograph of the droplets being moved along the channel. The larger width is again due to the heat affected zone, but the small difference in the refractive indices of the oil and PMMA makes the channels difficult to resolve. The smaller width is now the width taken up by the droplets.

1.2. Figure S2: Droplets In a Zig-Zag

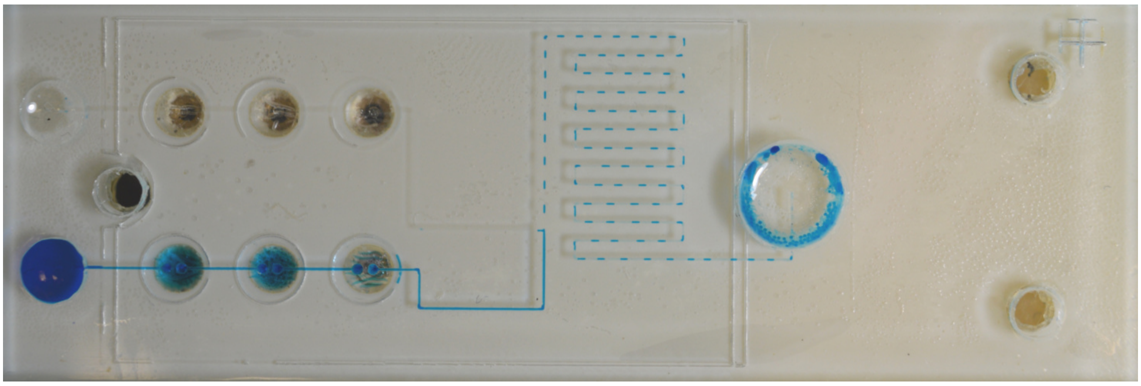

(a)

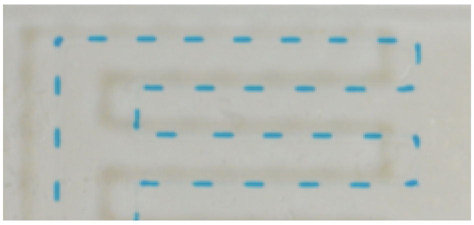

(b)

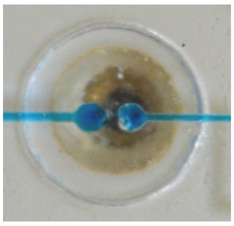

(c)

Figure 2. (a) Under the conditions of minimal backstep, the chip (Fig. 1c) generates a uniform train of droplets of water (blue) in oil (clear) that are drawn from the oil and water reservoirs on the left and marched through a complex structure to a waste well on the right. Not shown in Fig. 1c are the alignment holes (two on the right and one on the left). The chip is 76.2 x 25.4 mm and underneath are the 6 brass fittings screwed into the base of the chip, each connecting to an output from the pneumatic controller, which is in turn controlled via a laptop computer. **(b)** The top of the zig-zag structure is shown in higher resolution, with a shadow of the channel in the background. **(c)** Although the bottom right valve in (a) shows wrinkles in the membrane [from deformation under excessively large pressure differences] and some leakage into the moat around the valve, these defects are not apparent in the corresponding valve from a chip used only under the optimal pressure conditions. The brown shading is from the brass fitting that is attached underneath the chip.

© 2018 by the authors. Submitted to *Micromachines* for possible open access publication under the terms and conditions of the Creative Commons Attribution (CC BY) license (http://creativecommons.org/licenses/by/4.0/).
