# Supplementary figures and images for: CO2 Laser-Based Rapid Prototyping of Micropumps
Source: Micromachines (Basel). 2018 May 3;9(5):215. doi: 10.3390/mi9050215 (PMC6187535; doi:10.3390/mi9050215)

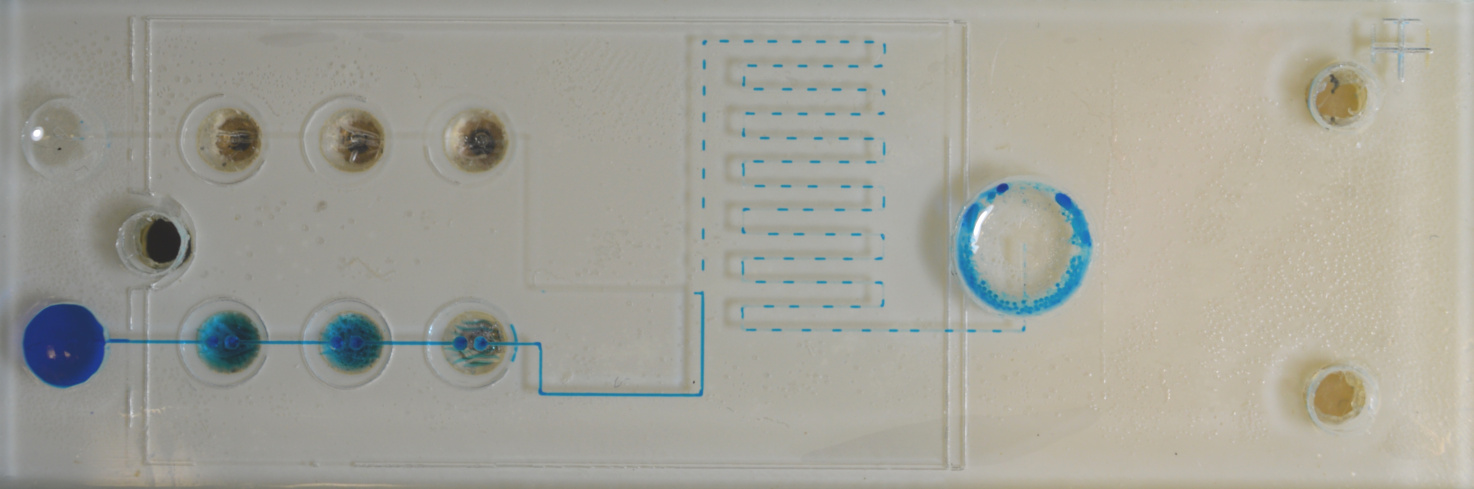

Supplement: Supplementary file 1 [file micromachines-09-00215-s001.zip › micromachines-284508-SI/Figures/FigS2a_DSC_0788small.jpg]

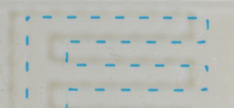

Supplement: Supplementary file 1 [file micromachines-09-00215-s001.zip › micromachines-284508-SI/Figures/FigS2b_DSC_0788small_TopZigZag.jpg]

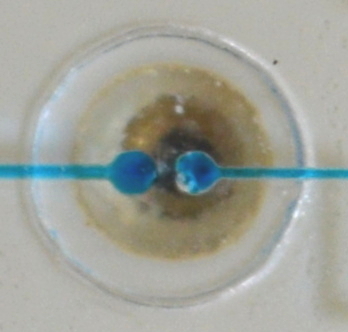

Supplement: Supplementary file 1 [file micromachines-09-00215-s001.zip › micromachines-284508-SI/Figures/FigS2c_FullChipNoWrinkles_2_BottomRightValve.jpg]

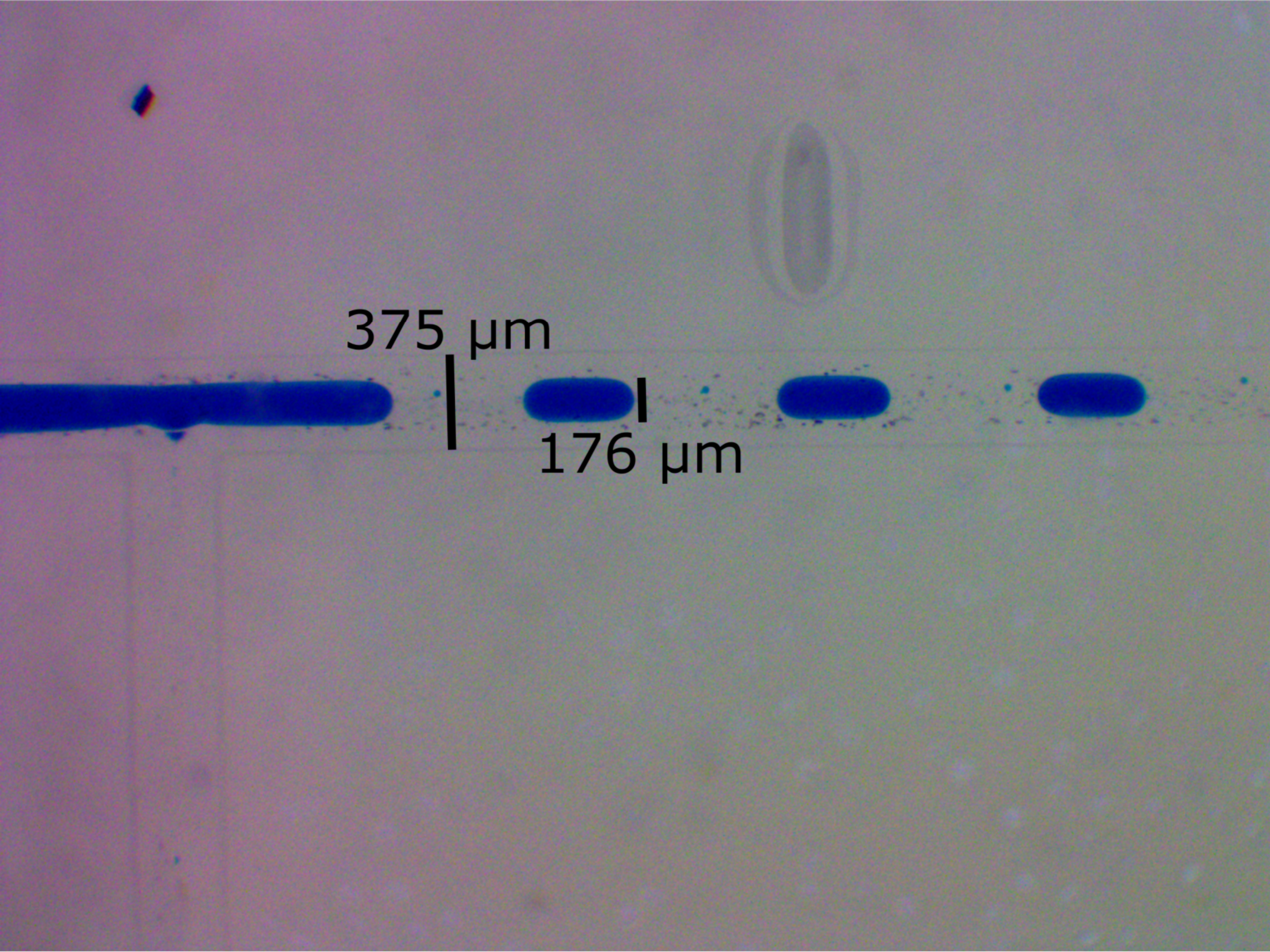

375  $\mu\text{m}$

176  $\mu\text{m}$

Supplement: Supplementary file 1 [file micromachines-09-00215-s001.zip › micromachines-284508-SI/Figures/S1b_DropsPair.pdf]

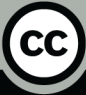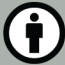

BY

Supplement: Supplementary file 1 [file micromachines-09-00215-s001.zip › micromachines-284508-SI/logo-ccby-eps-converted-to.pdf]

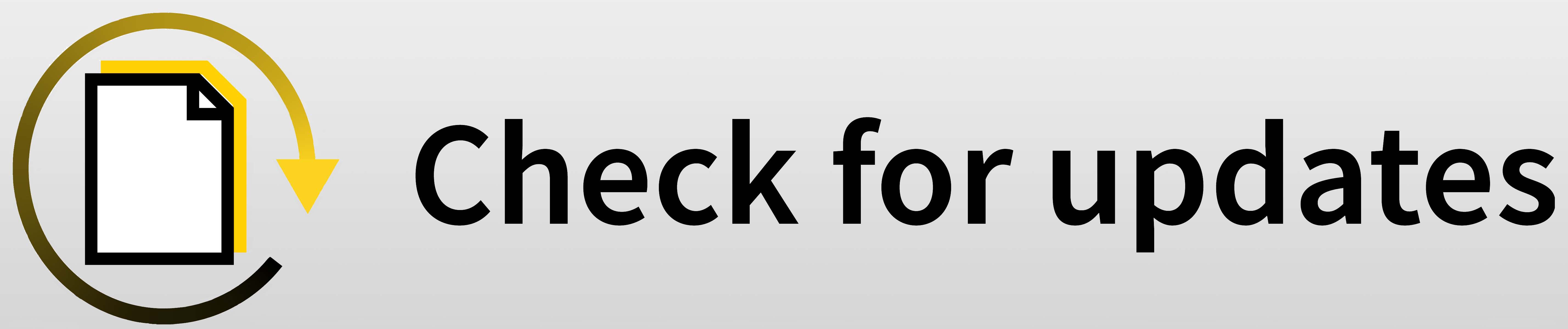

Supplement: Supplementary file 1 [file micromachines-09-00215-s001.zip › micromachines-284508-SI/logo-updates.jpg]
